# Increased platelet activation and lower platelet-monocyte aggregates in COVID-19 patients with severe pneumonia

**DOI:** 10.1371/journal.pone.0282785

**Published:** 2023-03-08

**Authors:** Sirada Srihirun, Thanaporn Sriwantana, Sirawat Srichatrapimuk, Pornpun Vivithanaporn, Suppachok Kirdlarp, Somnuek Sungkanuparph, Sithakom Phusanti, Nithita Nanthatanti, Prasit Suwannalert, Nathawut Sibmooh

**Affiliations:** 1 Department of Pharmacology, Faculty of Dentistry, Mahidol University, Bangkok, Thailand; 2 Chakri Naruebodindra Medical Institute, Faculty of Medicine Ramathibodi Hospital, Mahidol University, Samut Prakan, Thailand; 3 Department of Pathobiology, Faculty of Science, Mahidol University, Bangkok, Thailand; National Institutes of Health, National institute of Diabetes and Digestive and Kidney Diseases, UNITED STATES

## Abstract

**Background:**

The increased procoagulant platelets and platelet activation are associated with thrombosis in COVID-19. In this study, we investigated platelet activation in COVID-19 patients and their association with other disease markers.

**Methods:**

COVID-19 patients were classified into three severity groups: no pneumonia, mild-to-moderate pneumonia, and severe pneumonia. The expression of P-selectin and activated glycoprotein (aGP) IIb/IIIa on the platelet surface and platelet-leukocyte aggregates were measured prospectively on admission days 1, 7, and 10 by flow cytometry.

**Results:**

P-selectin expression, platelet-neutrophil, platelet-lymphocyte, and platelet-monocyte aggregates were higher in COVID-19 patients than in uninfected control individuals. In contrast, aGPIIb/IIIa expression was not different between patients and controls. Severe pneumonia patients had lower platelet-monocyte aggregates than patients without pneumonia and patients with mild-to-moderate pneumonia. Platelet-neutrophil and platelet-lymphocyte aggregates were not different among groups. There was no change in platelet-leukocyte aggregates and P-selectin expression on days 1, 7, and 10. aGPIIb/IIIa expression was not different among patient groups. Still, adenosine diphosphate (ADP)-induced aGPIIb/IIIa expression was lower in severe pneumonia than in patients without and with mild-to-moderate pneumonia. Platelet-monocyte aggregates exhibited a weak positive correlation with lymphocyte count and weak negative correlations with interleukin-6, D-dimer, lactate dehydrogenase, and nitrite.

**Conclusion:**

COVID-19 patients have higher platelet-leukocyte aggregates and P-selectin expression than controls, indicating increased platelet activation. Compared within patient groups, platelet-monocyte aggregates were lower in severe pneumonia patients.

## Introduction

The pandemic Coronavirus Disease 2019 (COVID-19) has affected people worldwide and manifests as acute respiratory tract symptoms, including flu-like symptoms, dyspnea, and pneumonia. Acute respiratory distress syndrome accounts for the majority of death among patients with severe pneumonia. In severe cases, COVID-19 can be complicated with life-threatening thrombosis, provoking organ failures such as pulmonary embolism, deep vein thrombosis, myocardial infarction, ischemic stroke, and acute limb ischemia [1, 2]. The arterial and venous circulations are both affected by COVID-19: 29.4% of intensive care unit (ICU) patients had a thrombotic event (13.6% venous and 18.6% arterial) [2]. The increased clotting activation in COVID-19 arises from immune dysregulation which is accompanied by cytokine storm and inflammation known to trigger coagulation and platelet activation [3].

Platelets of COVID-19 patients exhibit increased aggregation and expression of surface activation markers such as P-selectin in response to agonists [4–6], which are more evident in ICU patients. Platelet-leukocyte aggregates are elevated in COVID-19 patients [4, 6, 7]. Severe COVID-19 patients have excessive neutrophil activation, and platelet-neutrophil aggregates correlate with pulmonary disease severity [8]. Platelet-monocyte interaction leads to increased tissue factor expression by the monocytes [6]. However, lower platelet activity in non-severe COVID-19 patients was also reported [9]. In spite of elevated baseline platelet activation, the platelet hyporesponsiveness and platelet-leukocyte aggregates decline over time in fatal cases [10]. Several mechanisms can attribute to the increased platelet activation in COVID-19, including increased mitogen-activated protein kinase pathway and thromboxane production in platelets [4], direct platelet activation by viral spike protein [5], endothelial dysfunction with downregulated angiotensin-converting enzyme 2 [7, 11], and direct SARS-Cov-2 infection of endothelial cells [12].

Even though studies demonstrated the association of hemostatic activation with the pathogenesis of COVID-19 [13], the relevance of platelet activation with disease severity or clinical outcome is equivocal. Here, we investigated if platelet activation and platelet-leukocyte aggregates differed in patients with different severity. In addition, the correlations of platelet activation and platelet-leukocyte aggregates with markers of inflammation and thrombosis were tested.

## Materials and methods

### Subjects, blood collection, and measurement of blood chemistry

This study was approved by the Ramathibodi Hospital Ethics Committee (COA. MURA2020/819). The written informed consent was obtained by patients or their legal representatives per the Declaration of Helsinki. We recruited COVID-19 patients between May 2020 and December 2021 at Chakri Naruebodindra Medical Institute, Faculty of Medicine Ramathibodi Hospital, Mahidol University. The patients were SARS-CoV-2 positive by nasopharyngeal PCR test. Severe pneumonia was defined in critically ill patients with pneumonia diagnosed by computed tomography and requiring mechanical ventilation in ICU. Patients with mild-to-moderate pneumonia and without pneumonia were admitted to non-ICU wards. Blood samples were collected per routine testing prospectively on admission days 0, 7, and 10. The SARS-CoV-2-negative control group included subjects who never got the infection and had similar ages to the patient group.

Clinical parameters, including complete blood count, blood urea nitrogen, creatinine, total bilirubin, direct bilirubin, aspartate aminotransferase, alanine aminotransferase, and lactate dehydrogenase, were determined at the Pathological Laboratory of Chakri Naruebodindra Medical Institute. Plasma nitrite was measured by tri-iodide-based chemiluminescence [14] using the chemiluminescence NO analyzer (CLD88; EcoMedicsAG, Duernten, Switzerland). Soluble inflammatory and thrombotic markers were measured by flow cytometry using the Human Thrombosis Panel (LEGENDplex, BioLegend Inc. San Diego, CA).

### Measurement of platelet activation and platelet-leukocyte aggregates by flow cytometry

Flow cytometric assay of platelet activation and platelet-leukocyte aggregates was performed within three hours after blood collection. The citrated whole blood was diluted with PBS (1:10 v/v) and stained with PE-Cy5 labeled with anti-CD42b (BD bioscience, San Jose, CA) as a platelet marker, PE-labeled anti-CD62P (BD bioscience, San Jose, CA) or anti-P-selectin as a platelet degranulation marker and FITC-labeled anti-PAC1 (BD bioscience, San Jose, CA) that bound aGPIIb/IIIa. The samples were incubated in the dark at room temperature for 15 minutes and then fixed with 1% paraformaldehyde. Expression of P-selectin and aGPIIb/IIIa was measured using BD Accuri C6 Plus Flow Cytometer (BD bioscience, San Jose, CA). Platelets (10,000 events) were gated on the basis of light scatter and the expression of CD42b. The expression of P-selectin and aGPIIb/IIIa was defined as the percentage of positive events exhibiting fluorescence greater than the isotype controls. In separate experiments, the stained samples were incubated further with 1 μM ADP at room temperature for 15 minutes before being fixed with 1% paraformaldehyde.

To measure the platelet-leukocyte aggregates, we diluted the whole blood samples by10-fold with PBS and incubated them with FITC-labeled CD42a (a platelet marker), PE-labeled anti-CD62P (a platelet degranulation marker), PerCP-labeled CD45 (BD bioscience, San Jose, CA) and APC-labeled CD14 (BD bioscience, San Jose, CA) for 15 minutes in the dark at room temperature. FACS lysis buffer was added to lyzed red blood cells. Platelet-leukocyte aggregates were measured by BD Accuri C6 Plus Flow Cytometer (BD bioscience, San Jose, CA). The 15,000 CD45-positive events were collected as a leukocyte population. Neutrophils and lymphocytes were identified by the side scatter (SSC) and CD45 expression pattern, whereas monocytes were further confirmed by the expression of CD14. The events that exhibited fluorescence of CD42a and CD62P greater than isotype controls in the leukocyte subpopulation were identified as platelet-neutrophil, platelet-lymphocyte, and platelet-monocyte aggregates [15].

### Statistical analysis

Statistical analysis was performed by GraphPad-Prism version 4 (GraphPad Software Inc., San Diego, CA) using one-way ANOVA with Tukey’s multiple comparison test or Kruskal-Wallis test, depending on normality. The correlation was analyzed by Spearman’s rank test. A *p-value* of < 0.05 was considered statistically significant.

## Results

### Patients’ characteristics

Ninety-four COVID-19 patients and nine healthy control subjects were recruited (Table 1). The patients were admitted to our institute with severe pneumonia (n = 26) requiring intensive care, mild-to-moderate pneumonia (n = 36), and no pneumonia (n = 32). The patients with mild-to-moderate pneumonia (mean age of 47.1 years) and with severe pneumonia (mean age of 52.4 years) were older than those without pneumonia (mean age of 34.5 years) (by ANOVA, *p* < 0.05). Of all, 61.5% of severe patients were men. Severe pneumonia patients had a higher body mass index than patients without pneumonia. All severe pneumonia patients had risk factors for severe COVID-19 [16], including hypertension (38.5%), diabetes mellitus (50.0%), and obesity (23.1%). There was no difference in platelet number among the three groups on admission days 0, 7, and 10. On the day of admission, neutrophils, lymphocytes, blood urea nitrogen, and aspartate aminotransferase levels were higher in severe patients than in those with mild-to-moderate and without pneumonia. Creatinine and alanine aminotransferase were elevated in the severe group compared to the mild-to-moderate group. There was no difference in plasma nitrite.

**Table 1:** Clinical parameters of control and COVID-19 patients on the admission day.

	Control	COVID-19 patients			
		No pneumonia	Mild-to-moderate pneumonia	Severe pneumonia	Total
	(n = 9)	(n = 32)	(n = 36)	(n = 26)	(n = 94)
Age (mean ± SD), years	49.9 ± 17.5	34.5 ± 12.5	47.1 ± 13.6	52.4 ± 17.6^a^	44.3 ± 16.1
Male (%), n	2 (22.2)	14 (42.4)	18 (50.0)	16 (61.5)	48 (51.1)
Body mass index (mean ± SD)	24.2 ± 4.3	24.7 ± 4.6	27.1 ± 5.9	28.1 ± 6.6^b^	26.5 ± 5.8
**Cardiovascular risk factors, n (%)**					
Hypertension	2 (22.2)	4 (12.1)	5 (13.9)	10 (38.5)	19 (20.0)
Diabetes mellitus	0	4 (12.1)	4 (11.1)	13 (50.0)	21 (22.1)
Dyslipidemia	2 (22.2)	2 (6.1)	4 (11.1)	6 (23.1)	12 (12.6)
Atrial fibrillation	0	0 (0.0)	0 (0.0)	1 (3.8)	1 (1.1)
Coronary artery disease	0	1 (3.0)	0 (0.0)	0 (0.0)	1 (1.1)
Chronic kidney disease	0	2 (6.1)	0 (0.0)	3 (11.5)	5 (5.3)
Obesity	1 (11.1)	4 (12.1)	9 (25.0)	7 (26.9)	20 (21.1)
Current smokers	0	4 (12.1)	8 (22.2)	5 (19.2)	17 (17.9)
**Admission laboratory, median (IQR)**					
Hemoglobin, g/dL	13.7 (12.7–14.0)	14.2 (13.0–15.0)	13.6 (12.9–14.9)	13.0 (12.1–14.6)	13.7 (12.7–14.8)
Hematocrit, %	42.0 ± 1.9	43.5 ± 5.0	42.3 ± 4.7	39.3 ± 5.7	42.1 ± 5.4
Platelets, x 10^3^/μL	260.0 (244.0–285.0)	269.5 (236.3–317.3)	234.5 (194.3–292.5)	246.0 (206.8–318.5)	258.0 (205.3–298.8)
Leukocytes, x 10^3^/μL	6.6 (5.2–9.4)	6.2 (5.4–7.6)	5.6 (4.2–6.6)	8.7 (6.1–12.2)	6.2 (4.8–7.8)
Neutrophils, x 10^3^/μL	4.0 (2.9–6.2)	3.1 (2.2–4.4)	3.0 (2.3–3.9)	7.8 (4.8–10.9)^a^	3.5 (2.5–5.1)
Lymphocytes, x 10^3^/μL	2.2 (1.8–2.9)	2.3 (1.9–3.2)	1.6 (1.4–2.5)	0.75 (0.6–1.1)^a^	1.7 (1.1–2.5)
Monocytes, x 10^3^/μL	0.3 (0.2–0.4)	0.39 (0.3–0.5)	0.4 (0.3–0.5)	0.3 (0.2–0.5)	0.4 (0.3–0.5)
Eosinophil, x 10^3^/μL	0.04 (0.03–0.05)	0.1 (0.06–0.17)	0.07 (0–0.12)	0 (0–0)	0.1 (0.1–0.7)
Basophil, x 10^3^/μL	0.5 (0.4–0.7)	0 (0–0.06)	0 (0–0.05)	0 (0–0)	0 (0–0.05)
Blood urea nitrogen, mg/dL	13.0 (11.5–16.3)	12.0 (10.0–14.0)	11.0 (10.0–13.0)	20.0 (15.7–26.3)^a^	13.0 (11.0–16.0)
Creatinine, mg/dL	0.6 (0.6–0.8)	0.8 (0.7–1.0)	0.8 (0.8–1.0)	0.9 (0.8–1.2)^c^	0.8 (0.7–1.0)
GFR, mL/m^2^	NA	106.1 (96.6–119.8)	95.1 (82.2–104.1)	88.2 (54.5–104.1)	98.5 (83.2–110.3)
Total bilirubin, mg/dL	NA	0.5 (0.4–0.8)	0.5 (0.4–0.6)	0.5 (0.3–0.6)	0.5 (0.4–0.6)
Direct bilirubin, mg/dL	NA	0.2 (0.2–0.2)	0.2 (0.2–0.3)	0.2 (0.2–0.3)	0.2 (0.2–0.3)
AST, U/L	21.0 (19.5–23.0)	21.0 (12.5–29.5)	25.0 (21.0–29.5)	54.5 (30.0–103.3)^a^	26.0 (21.0–33.0)
ALT, U/L	21.5 (12.3–29.3)	23.0 (16.0–36.0)	25.0 (17.3–39.3)	45.5 (23.8–95.5)^c^	28.0 (18.0–42.0)
LDH, U/L	NA	168.5 (152.0–180.5)	192.5 (161.5–230.3)	342.0 (314.8–496.5)^a^	195.5 (165.3–291.3)
Plasma nitrite, nM	9.4 (5.2–30.7)	8.66 (5.9–14.3)	10.8 (5.2–16.1)	13.6 (7.4–33.3)	10.93 (6.5–17.1)

ALT, alanine aminotransferase; AST, aspartate aminotransferase; IQR, interquartile range; GFR, glomerular filtration rate; LDH, lactate dehydrogenase; SD, standard deviation.

^a^*p* < 0.05 compared with no pneumonia and mild-to-moderate pneumonia groups

^b^*p* < 0.05 compared with no pneumonia group

^c^*p* < 0.05 compared with mild-to-moderate pneumonia group

### The P-selectin expression on platelets

**Fig 1:**
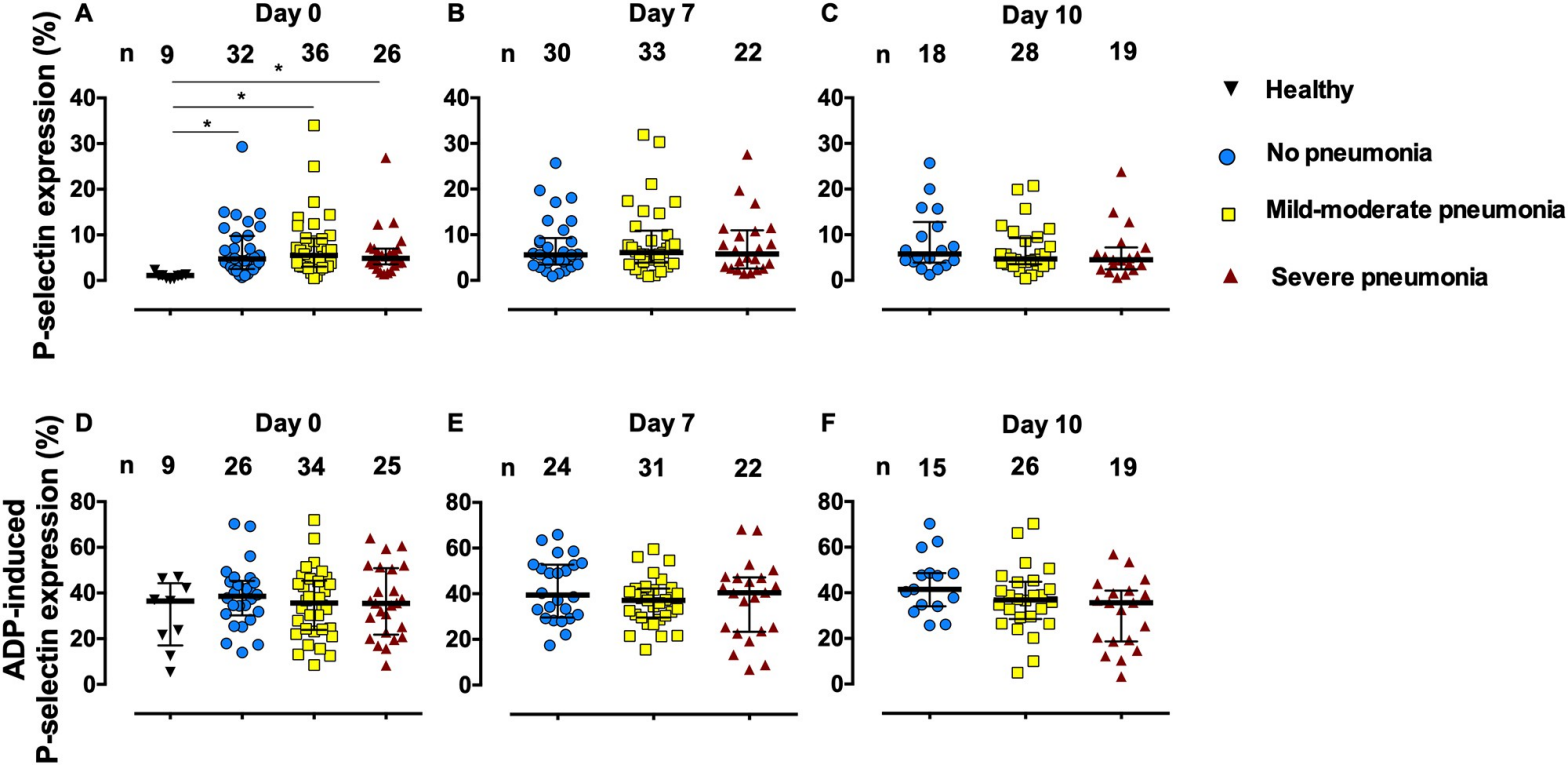
The P-selectin expression on platelets without (A-C) and with 1 μM ADP stimulation (D-F) in healthy control subjects and COVID-19 patients on admission days 0, 7, and 10. Lines represent medians and interquartile ranges.

The baseline P-selectin expression was higher in COVID-19 patients than in controls (Fig 1A). COVID-19 patients with no pneumonia, mild-to-moderate pneumonia, and severe pneumonia had higher baseline platelet P-selectin expression than controls on admission days 0, 7, and 10 (Fig 1A–1C). No difference in P-selectin expression was observed among the three severity groups. There was no change in P-selectin expression on days 0, 7, and 10 when compared within the severity group.

P-selectin expression in response to ADP (1 μM) stimulation was not different compared between each COVID-19 severity group and controls (Fig 1D–1F). No difference in ADP-induced P-selectin expression was seen among the severity groups. There was no change in ADP-induced P-selectin expression on days 0, 7, and 10 when compared within the severity group.

### aGPIIb/IIIa expression on platelets

**Fig 2:**
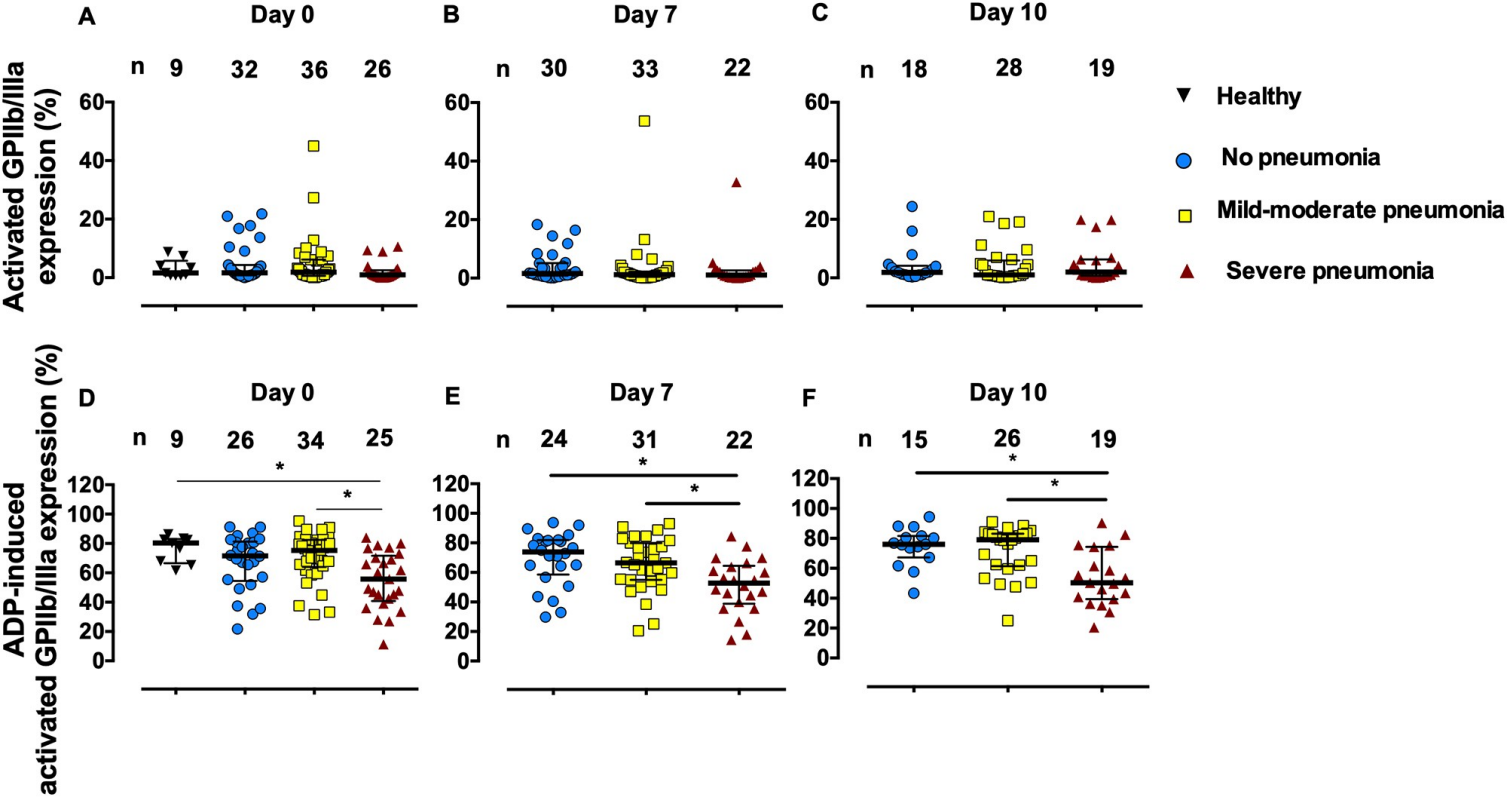
Activated glycoprotein (GP) IIb/IIIa expression on platelets without (A-C) and with 1 μM ADP stimulation (D-F) in healthy control subjects and COVID-19 patients on admission days 0, 7, and 10. Lines represent medians and interquartile ranges. *p < 0.05 by Kruskal-Wallis test.

The baseline aGPIIb/IIIa expression in COVID-19 patients was not different from the values in controls (Fig 2A–2C). There was no difference in ADP (1 μM)-induced aGPIIb/IIIa expression between COVID-19 and control groups (Fig 2D–2F). On admission day 0, platelets from patients with severe pneumonia exhibited lower ADP-induced aGPIIb/IIIa expression than those from patients with mild-to-moderate pneumonia (median (IQR): 55.80 (40.75–71.65) vs. 75.25 (63.98–82.43)%, *p* = 0.0067). On days 7 and 10, the lower ADP-induced aGPIIb/IIIa expression in patients with severe pneumonia was seen compared with those without pneumonia and with mild-to-moderate pneumonia.

### Platelet-leukocyte aggregates

**Fig 3:**
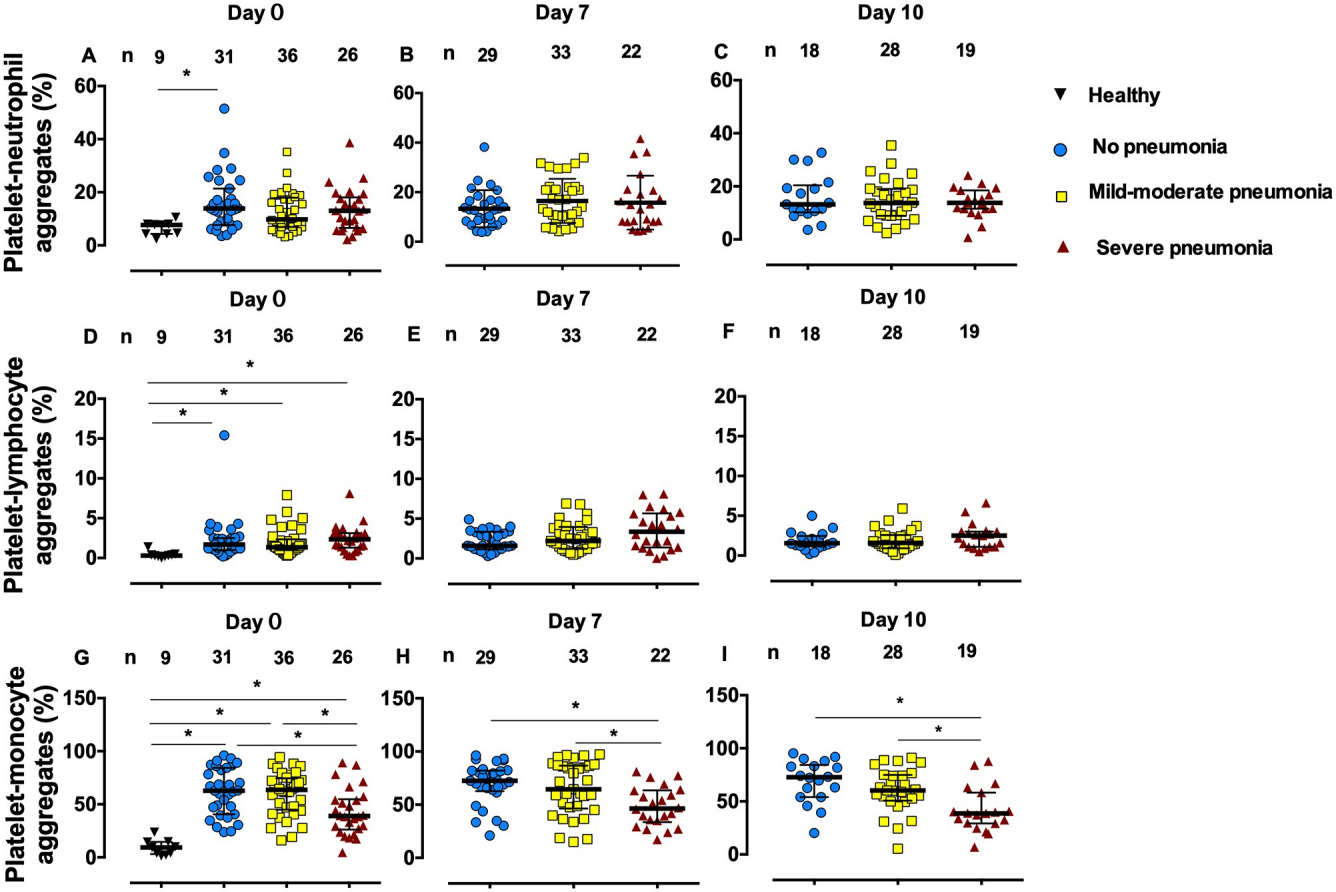
Platelet-neutrophil aggregates (A-C), platelet-lymphocyte aggregates (D-F), and platelet-monocyte aggregates (G-I) in healthy control subjects and COVID-19 patients on admission days 0, 7, and 10. Lines represent medians and interquartile ranges. *p < 0.05 by Kruskal-Wallis test.

COVID-19 patients had higher platelet-neutrophil, platelet-lymphocyte, and platelet-monocyte aggregates than controls (Fig 3). There were no differences in platelet-neutrophil and platelet-lymphocyte aggregates compared within COVID-19 groups (Fig 3A–3F). In COVID-19 groups, the platelet-neutrophil and platelet-lymphocyte aggregates did not change when compared among admission days 0, 7, and 10. Nonetheless, platelets from patients with severe pneumonia showed decreased platelet-monocyte aggregates compared with patients with mild-to-moderate pneumonia and without pneumonia on admission days 0, 7, and 10 (Fig 3G–3I). On admission day 0, platelet-monocyte aggregates were lower in the severe pneumonia group than in mild-to-moderate pneumonia and without pneumonia groups (median (IQR): 38.90 (26.18–54.75), 63.60 (44.58–74.53), and 62.82 (40.60–84.30)%, respectively, *p* = 0.0035).

### Soluble inflammatory and thrombotic markers

On admission day, patients with severe pneumonia had elevated soluble markers of inflammation and thrombosis (Table 2). IL-6, soluble P-selectin, P-selectin glycoprotein ligand-1, tissue plasminogen activator, plasminogen activator inhibitor, tissue factor, and D-dimer increased in patients with severe pneumonia than those with mild-to-moderate pneumonia and without pneumonia. Although there was no difference in plasma nitrite (a marker of nitric oxide formation) among the groups, nitrite correlated inversely with baseline aGPIIb/IIIa expression (*r* = -0.314, *p* = 0.010), ADP-induced aGPIIb/IIIa expression (*r* = -0.294, *p* = 0.021), and platelet-monocyte aggregates (*r* = -0.356, *p* = 0.003).

**Table 2:** Soluble inflammatory and thrombotic markers on the admission day.

Markers	No pneumonia	Mild-to-moderate pneumonia	Severe pneumonia
Interleukin-6, pg/mL	4,447 (4,195–5,118)	5,075 (4,394–5,769)	5,680 (5,167–6,428)*
Interleukin-8, pg/mL	3,950 ± 1,174	3,953 ± 1,210	4,736 ± 1,246
Soluble P-selectin, pg/mL	6,204 (5,203–7,461)	6,504 (5,315–7,475)	8,369 (6,598–10,378)*
P-selectin glycoprotein ligand-1, pg/mL	8,481 (7,886–8,846)	9,190 (8,557–9,603)	10,052 (9,826–10,319)*
CD40L, pg/mL	9,035 ± 1,056	9,083 ± 899.1	9,540 ± 1,291
Tissue plasminogen activator, pg/mL	14,603 (12,557–17,539)	19,253 (16,258–25,178)^#^	25,793 (23,896–37,323)*
Plasminogen activator inhibitor, pg/mL	15,320 (13,728–18,931)	18,477 (16,051–21,961)	21,642 (18,822–26,892)*
Tissue factor, pg/mL	5,314 (4,995–5,910)	5,470 (5,057–6,039)	5,933 (5,791–6,183)*
Factor IX, pg/mL	28,425 (22,713–38,708)	32,932 (27,800–42,198)	30,156 (26,635–34,429)
D-dimer, ng/mL	1,724 (1,587–2,148)	2,002 (1,820–2,327)	2,583 (2,279–2,764)*

Data are means ± standard deviation or medians (interquartile range), depending on normality.

*p < 0.05 compared with no pneumonia and mild-moderate pneumonia

### Correlation

**Fig 4:**
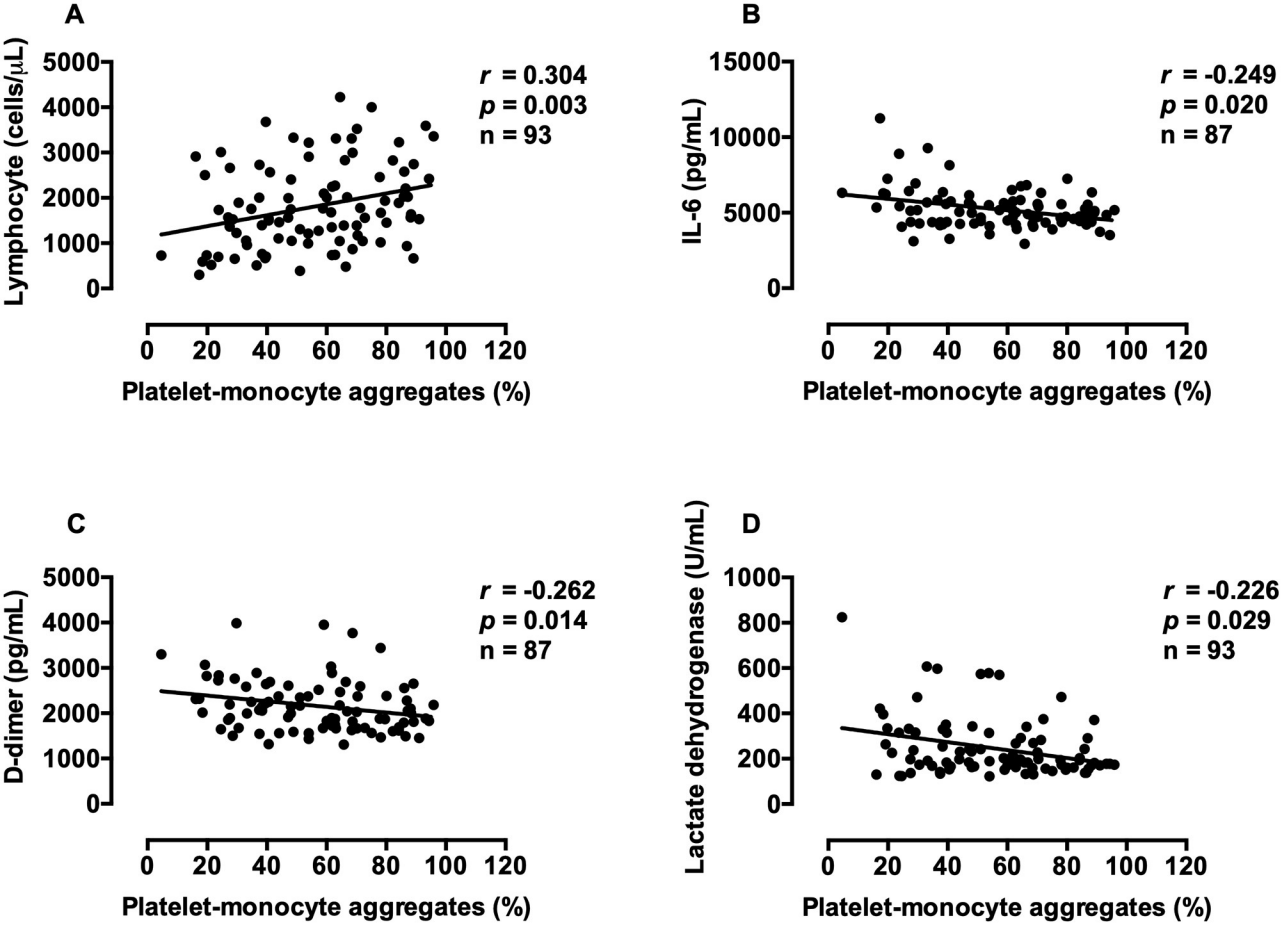
Correlations of platelet-monocyte aggregates with lymphocyte count, interleukin-6, D-dimer, and lactate dehydrogenase were analyzed by Spearman’s rank test.

To test the potential clinical association of our findings, we analyzed the correlations of platelet-monocyte aggregates with lymphocyte count, interleukin (IL)-6, D-dimer, and lactate dehydrogenase at the days of admission. Platelet-monocyte aggregates positively correlated with lymphocytes and negatively correlated with IL-6, D-dimer, and lactate dehydrogenase (Fig 4). Weak negative correlations were also observed for ADP-induced aGPIIb/IIIa expression with IL-6 (*r* = -0.220, *p* = 0.047) and D-dimer (*r* = -0.243, *p* = 0.028). There was no correlation between ADP-induced aGPIIb/IIIa expression versus platelet-monocyte aggregates of COVID-19 patients on day 0 (S1 Fig).

## Discussion

Our observational findings of increased platelet activation and platelet-leukocyte aggregates in COVID-19 are consistent with previous publications [4–7]. The P-selectin expressions at the basal state and in response to ADP stimulation were higher in COVID-19 patients than in controls. The patients also had higher platelet-leukocyte aggregates than controls. Additionally, we provide information that patients with severe pneumonia had lower platelet-monocyte aggregates, on admission days 0, 7, and 10, than patients without pneumonia and with mild-to-moderate pneumonia. The platelet-leukocyte aggregates did not change on admission days 0, 7, and 10. Although the aGPIIb/IIIa expression did not change in COVID-19 patients compared to controls, the lower aGPIIb/IIIa expression in response to ADP stimulation suggested platelet exhaustion in severe pneumonia patients.

Our results confirm the previous reports regarding dysregulated platelet activity in COVID-19 [4, 8, 10]. Increased baseline platelet activity and hyporesponsiveness to agonists in severe COVID-19 patients were reported previously [10]. The baseline P-selectin expression increased in COVID-19 patients. However, the ADP-induced P-selectin expression was not different between the patients and control subjects for an unknown reason. Varying ADP concentrations are required. In fatal cases, platelet-monocyte and platelet-neutrophils aggregates decreased over time, and the platelet responsiveness to ADP and thrombin receptor activator peptide 6 was reduced in the worsening patients [10]. Here, we provide more evidence, particularly the reduced platelet-monocyte aggregates and ADP-induced aGPIIb/IIIa expression.

Platelets show increased reactivity during viral infections [17, 18], including SARS-Cov-2 [13]. Platelet activation is accompanied by adhesion, aggregation, and secretion. Not all aspects of platelet response were elevated in our patients; the baseline P-selectin expression (degranulation marker) was higher, but aGPIIb/IIIa expression (aggregation marker) was not changed. Indeed, aGPIIb/IIIa expression was found to be reduced in COVID-19 [4] and influenza [19], suggesting that aGPIIb/IIIa expression does not increase in viral infection.

Many mechanisms account for the increased platelet activation in COVID-19, including the increased circulating proinflammatory and procoagulant mediators that can activate platelets. Exposure of platelets and whole blood from healthy subjects to plasma of severe COVID-19 patients resulted in platelet activation, platelet-leukocyte aggregate formation, and tissue factor expression on platelets, which were inhibited by tocilizumab, an IL-6 receptor neutralizing antibody [6, 7, 20]. Internalization of SARS-CoV or binding and activation of angiotensin-converting enzyme 2 by the viral spike protein can lead to platelet activation through the downstream mitogen-activated protein kinases pathway [5, 21].

Moreover, endothelial dysfunction, as shown by low plasma arginine and high asymmetric dimethylarginine (an endogenous nitric oxide synthase inhibitor) in COVID-19 patients [7, 22], can be responsible for increased platelet activation. The plasma nitric oxide metabolites, including nitrite and S-nitrosothiol, were reduced in COVID-19 patients due to endotheliitis and decreased synthesis; however, the patients who died had higher nitric oxide than the patients who survived [23]. We found no difference in plasma nitrite concentrations between controls and COVID-19 patients. However, a trend toward an increase in nitrite concentrations in the severe group was observed, which could be explained by the increased activity of inducible nitric oxide synthase, an enzyme expressed in inflammatory cells, in severe patients [24].

Platelet-leukocyte aggregates increase in COVID-19 patients [4, 6], which is also observed in our study. Apart from hemostasis, platelets can modulate leukocyte activities during infection. As thrombo-inflammatory markers, platelet-leukocyte aggregates are elevated in infectious diseases, cardiovascular diseases, stroke, lung inflammation, and hematologic diseases [13, 25, 26]. Platelet activation leads to receptor expression and secretion of mediators that facilitate platelet-leukocyte interactions. For example, P-selectin on platelets binds to the P-selectin glycoprotein ligand on neutrophils, forming platelet-neutrophil aggregates and releasing neutrophil extracellular traps [13]. No change in platelet-neutrophil aggregates in our study may be explained by their accumulation in pulmonary microcirculation [27]. The platelet-leukocyte interactions trigger mutual activation and mediator release from platelets and leukocytes.

Increased platelet-monocyte aggregates in severe COVID-19 patients were associated with tissue factor expression in monocytes by the mechanism involving P-selectin and GPIIb/IIIa complex [6]. From our data, all groups of COVID-19 patients showed higher platelet-monocyte aggregates than controls. In our study, the platelet-monocyte aggregates on day 30 in one severe patient were 80% which was still higher than the values of healthy subjects. Platelet hyperactivity in COVID-19 patients could persist up to 78 days after the onset of symptoms [28].

Within the COVID-19 patients, the patients with severe pneumonia had decreased platelet-monocyte aggregates compared to those without pneumonia and with mild-to-moderate pneumonia. The mechanism and impact of decreased platelet-monocyte aggregates have not been studied. Together with the reduction in ADP-induced aGPIIb/IIIa expression in severe patients, the decreased platelet-monocyte aggregates in severe cases may be explained by platelet exhaustion and increased platelet apoptosis in COVID-19 [29]. The decreased platelet-monocyte aggregates in severe patients were not related to monocyte count changes, as there was no association between the decreased CD14^+^ (a monocyte marker) cells and platelet-monocyte aggregates (S2 and S3 Figs). Further study is required to elucidate the mechanism and implication of decreased platelet-monocyte aggregates in severe pneumonia patients.

D-dimer is a fibrin degradation product used clinically as a marker of ongoing thrombosis. Abnormal coagulation indicated by elevated D-dimer is associated with the progression of thrombosis and pulmonary embolism. Elevated D-dimer is present in COVID-19 and associated with disease severity [30]. We found that patients with severe pneumonia had increased D-dimer compared to patients without pneumonia or with mild-to-moderate pneumonia, and the D-dimer levels negatively correlated with platelet-monocyte aggregates. Thus, patients with lower platelet-monocyte aggregates might have a higher risk of thrombosis and severe pneumonia. In addition, our results showed that other severity markers were associated with platelet-monocyte aggregates, including lymphocyte count [31], lactate dehydrogenase, and IL-6 [32].

There were limitations in our study. We did not identify the underlying mechanism of the decrease in platelet-monocyte aggregates and ADP-induced aGPIIb/IIIa expression, potentially including platelet exhaustion, derangement, apoptosis during SARS-Cov-2 infection [29], and unidentified plasma components [10]. A larger study is warranted to assess the power of platelet-monocyte aggregates as a marker of disease severity. In summary, platelets of COVID-19 patients have increased activation with lower platelet-monocyte aggregates in severe cases. Platelet-monocyte aggregates are promising to be a severity marker in COVID-19.

## Conclusion

In summary, we found that platelets of COVID-19 patients exhibited a hyperactivation state, as shown in increased baseline P-selectin expression and platelet-leukocyte aggregates. The severe patients had lower platelet responsiveness to ADP and platelet-monocyte aggregates. Platelet-monocyte aggregates correlate with disease markers.

## Supporting information

S1 Fig The plot of ADP-induced aGPIIb/IIIa expression versus platelet-monocyte aggregates of COVID-19 patients on admission day 0. The correlation was analyzed by Spearman’s rank test.
(TIFF)

S2 Fig The number of CD14^+^ events gated by flow cytometry. (TIFF)

S3 Fig The plot of CD14^+^ events versus platelet-monocyte aggregates. The correlation was analyzed by Spearman’s rank test.
(TIFF)

S1 Table Raw data of clinical parameter of control and COVID-19 patients on admission day. (XLSX)

S2 Table Raw data of platelet activation and platelet-leukocyte aggregates. (XLSX)

S3 Table Raw data of soluble inflammatory and thrombotic markers in COVID-19 patients on admission day. (XLSX)
