# Supplementary figures and images for: Increased platelet activation and lower platelet-monocyte aggregates in COVID-19 patients with severe pneumonia
Source: PLoS One. 2023 Mar 8;18(3):e0282785. doi: 10.1371/journal.pone.0282785 (PMC9994685; doi:10.1371/journal.pone.0282785)

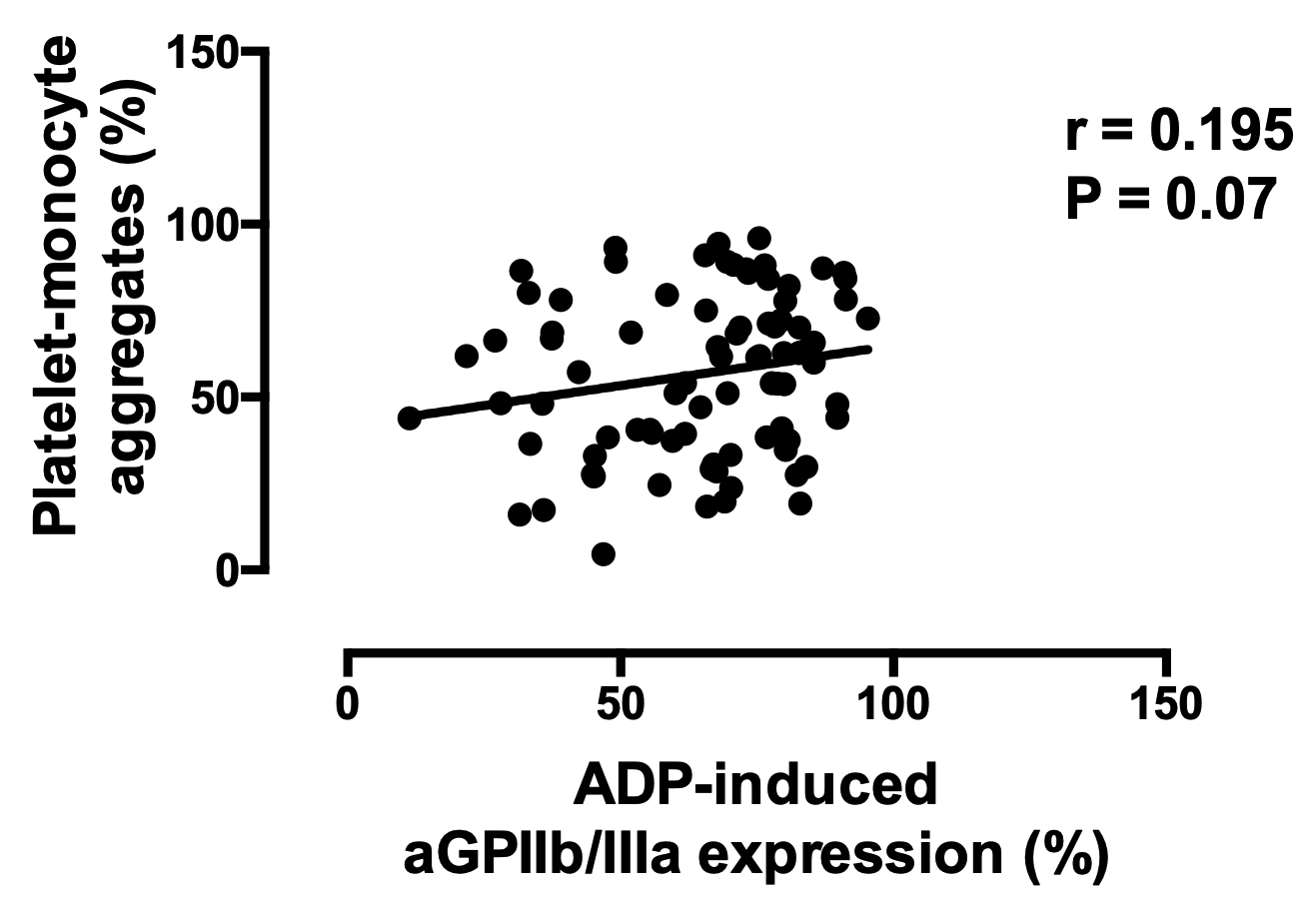

Supplement: S1 Fig — The correlation was analyzed by Spearman’s rank test. (TIFF) [file pone.0282785.s001.tiff]

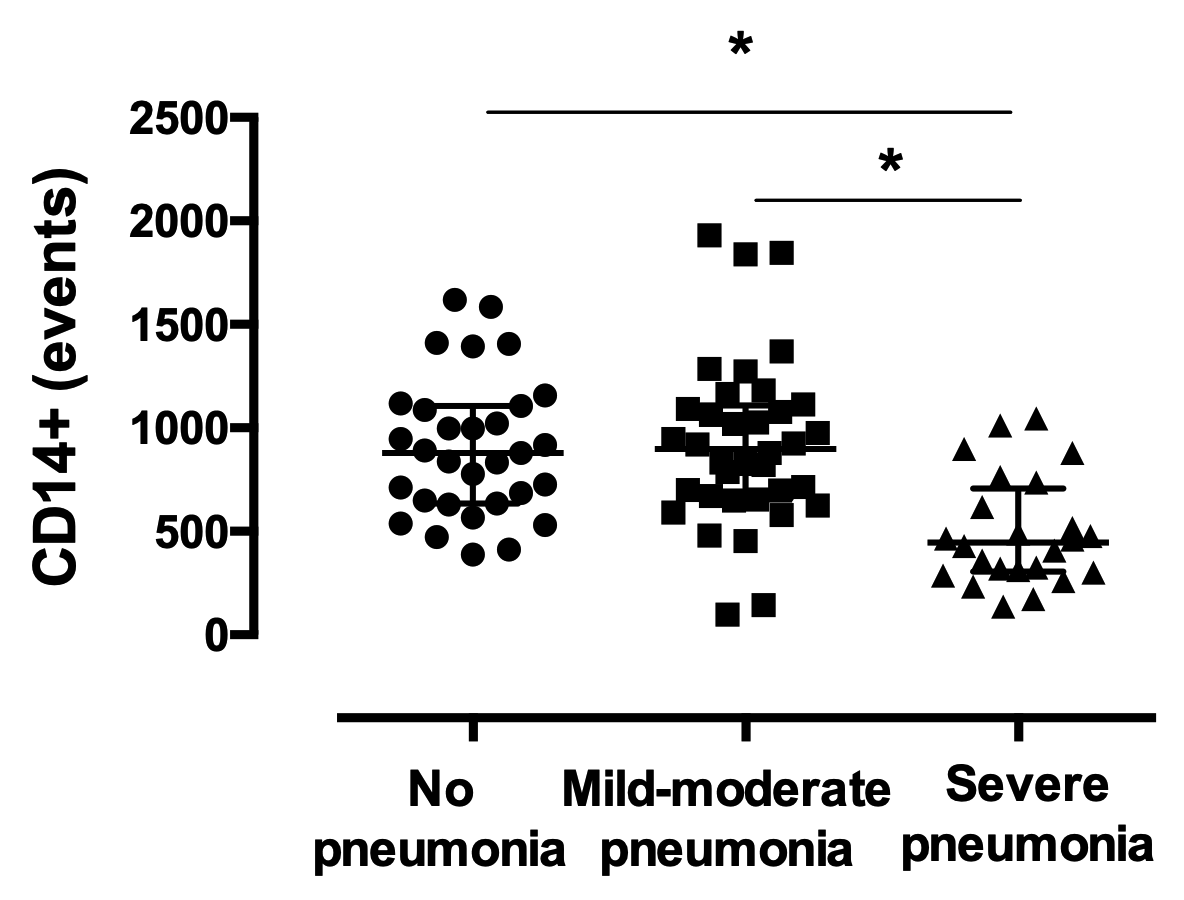

Supplement: S2 Fig — (TIFF) [file pone.0282785.s002.tiff]

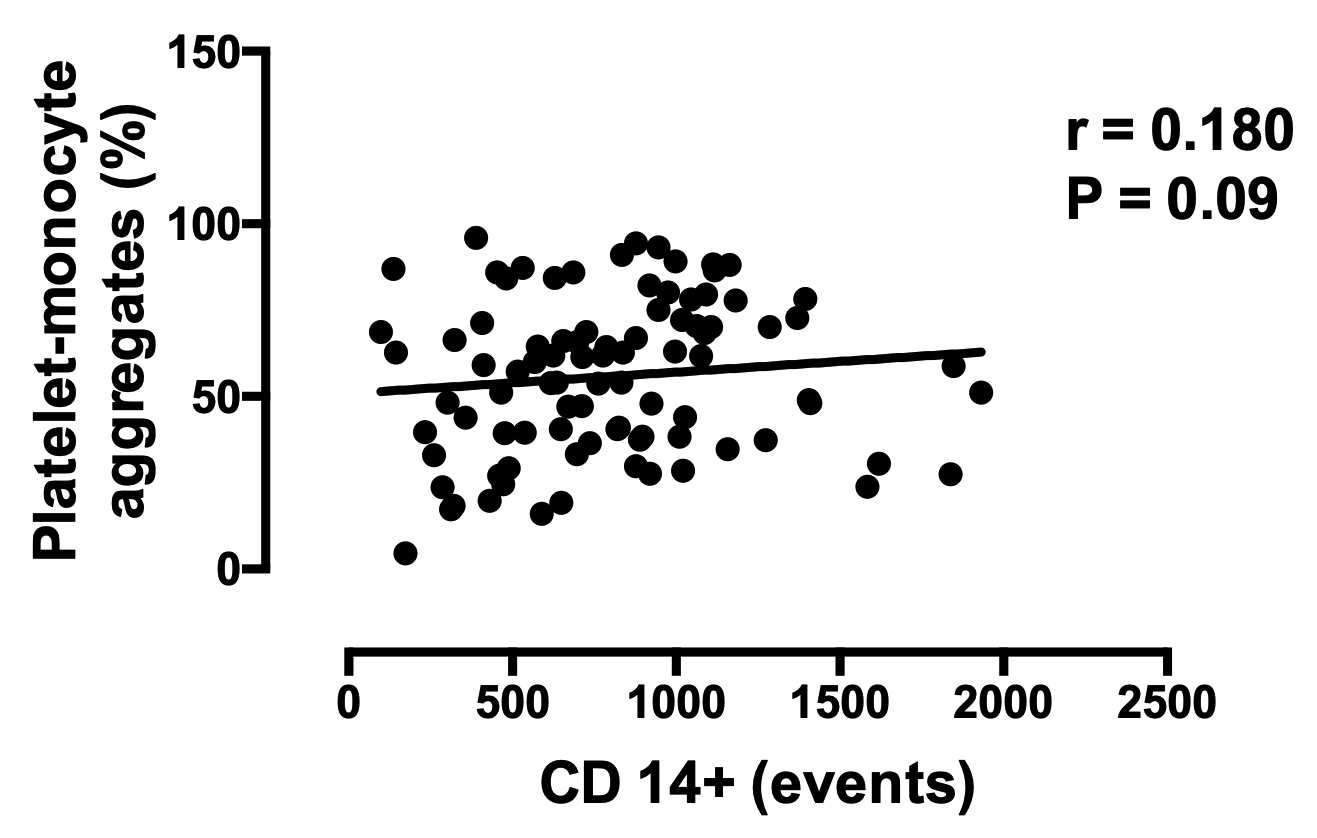

Supplement: S3 Fig — The correlation was analyzed by Spearman’s rank test. (TIFF) [file pone.0282785.s003.tiff]
